# Gastrocnemius release is an effective management option for Achilles tendinopathy: a systematic review

**DOI:** 10.1007/s00167-022-07039-7

**Published:** 2022-07-11

**Authors:** Zaki Arshad, Sofyan Al Shdefat, Adil M. Iqbal, Maneesh Bhatia

**Affiliations:** 1 School of Clinical Medicine, University of Cambridge, Cambridge, CB2 0SP UK; 2 Department of Trauma and Orthopaedic Surgery, University Hospitals of Leicester NHS Trust, Leicester, UK

**Keywords:** Achilles tendinopathy, Gastrocnemius recession, Gastrocnemius release, Systematic review

## Abstract

**Purpose:**

This systematic review aims to summarise the outcomes of gastrocnemius release in the management of Achilles Tendinopathy.

**Methods:**

A systematic review was performed according to PRISMA guidelines. A computer-based search was performed in PubMed, Embase, Cinahl, Scopus and ISI Web of Science. Two independent reviewers performed both title/abstract and full-text screening according to a-priori selection criteria. English-language original research studies reporting outcomes for gastrocnemius recession in patients with Achilles tendinopathy were included. Study quality and risk of bias were assessed using the MINORS criteria.

**Results:**

Of the 229 articles identified following database searching, nine studies describing 145 cases of gastrocnemius recession were included in the review. Clinically important differences were reported across a range of validated patient reported outcome scores including VISA-A, FFI, FAAM and VAS pain score. Outcomes appear to be superior in patients with noninsertional Achilles tendinopathy, however further research is required to confirm this. Studies also reported an increase in ankle dorsiflexion range of motion and a good rate of return to sport/work. The overall complication rate was 10/123 (8.1%), with sural nerve injury being the most common complication, occurring in 5/123 (4.1%) of cases.

**Conclusion:**

The results of this review suggest gastrocnemius release to be an effective treatment option in the management of patients with Achilles tendinopathy, who have gastrocnemius contracture and have previously failed to respond adequately to non-operative treatment.

**Level of evidence:**

Level IV.

## Introduction

Achilles tendinopathy (AT) occurs in approximately 1.85 per 1000 people and presents with functional restriction, pain and swelling at the calcaneal insertion (insertional AT) or 2–6 cm proximal to the calcaneal insertion (non-insertional AT) [9, 19, 24, 26]. While the exact aetiology of AT is not clear, it is believed to involve degeneration or failed adaptation of the Achilles tendon to activity [9, 10, 24].

First line management for AT is non-operative; however, after 6 months of non-operative treatment, up to 45.5% of patients may remain symptomatic and undergo operative intervention [1, 9, 21, 28, 41, 43]. Surgical strategies may include: debridement of fibrotic adhesions and regions of failed healing, controlled longitudinal incisions to the tendon to restore vascularity and stimulate a healing response, graft augmentation and removal of neovessels and associated nerves [25, 31, 34]. These surgical approaches are often effective, with research suggesting a success rate of over 80% [25].

AT, as well as other foot and ankle conditions such as plantar fasciitis, have been linked to gastrocnemius contracture [3]. It is thought that this contracture limits dorsiflexion of the ankle and places increased strain on the Achilles tendon, calcaneal tuberosity and plantar fascia [3, 8, 46]. It, therefore, logically follows that reducing tension in the gastrocnemius through a recession procedure may be useful in the management of these conditions. While some studies do show promising results following the use of gastrocnemius recession in patients with AT, use of this technique is still debated and yet to gain widespread recognition as a potential treatment option in this clinical scenario [14, 15, 27].

This comprehensive systematic review aims to address this uncertainty by evaluating the role of gastrocnemius recession in the management of AT, with respect to potential outcomes and peri-operative complications. Through this, clinicians will be provided with an improved understanding of the role of the procedure in the management of AT.

## Materials and methods

The PRISMA guidelines for systematic reviews and meta-analyses were followed in the conductance of this review [39].

### Search strategy

A comprehensive computer-based search was performed in five electronic databases including, PubMed, Embase, Cinahl, Scopus and ISI Web of Science. All databases were searched from inception to 7th January 2022. The search strategy involved the terms ‘gastrocnemius’, ‘recession’, ‘lengthening’, ‘release’, ‘Achilles’, ‘Tendinopathy’ and ‘tendinitis’, combined with the Boolean Operators (AND, OR), where appropriate. All searches were performed using an English Language filter.

### Data management

All studies retrieved following the described searching processes were imported into Rayyan systematic review web application tool, which was used to facilitate study screening [38].

### Selection process and criteria

A two-stage title/abstract and full-text screening was performed according to the review selection criteria, which were established a-priori. Differences in opinion at either selection stage were resolved first by discussion between the two reviewers and failing this, by consultation with a third senior reviewer. The selection criteria displayed according to the PICOS framework are as follows.

*Participants*: Patients of any age with insertional or non-insertional Achilles tendinopathy.

*Intervention*: Studies describing gastrocnemius recession only were included. Articles reporting gastrocnemius recession with concomitant procedures such as Achilles tendon lengthening were excluded.

*Control*: No control group was required for inclusion in this review.

*Outcomes*: Studies describing any patient reported outcome measure (PROM), such as the American Orthopaedic Foot and Ankle Society (AOFAS) score, visual analogue score (VAS) for pain Victorian Institute of Sports Assessment (VISA-A) questionarie, Foot and Ankle Ability Measure (FAAM), Foot function index (FFI)and short form-36 health survey (SF-36) were included. Secondary outcomes included, ankle range of motion, gastrocnemius power return to sport/work and complications.

*Study design:* Original research studies (randomised control trials, case control studies, cohort studies and case series) were included. Conference abstracts, review articles, commentaries, letters to the editor and case reports were excluded.

Due to the limited translation ability of the review team, only studies published in the English language were included. No date of publication restrictions was imposed for inclusion in the review. Some studies included patients with different conditions, all receiving gastrocnemius recession. These were only included if it was possible to separate the results to specifically isolate the patients with AT receiving gastrocnemius recession.

### Data extraction

Data were extracted by two authors using an extraction spreadsheet created on Microsoft Excel with the following headings: (1) author, (2) publication year, (3) title, (4) study type, (5) number of patients/feet undergoing gastrocnemius recession for AT, (6) age, (7) male:female ratio, (8) insertional or non-insertional AT, (9) endoscopic or open gastrocnemius recession, (10) follow-up period, (11) patient-reported outcome scores, (12) VAS pain score, (13) association between patient/treatment-related factor any outcome (14) complications, (15) ankle range of motion, and (16) return to work/sport.

### Data synthesis

**Fig. 1:**
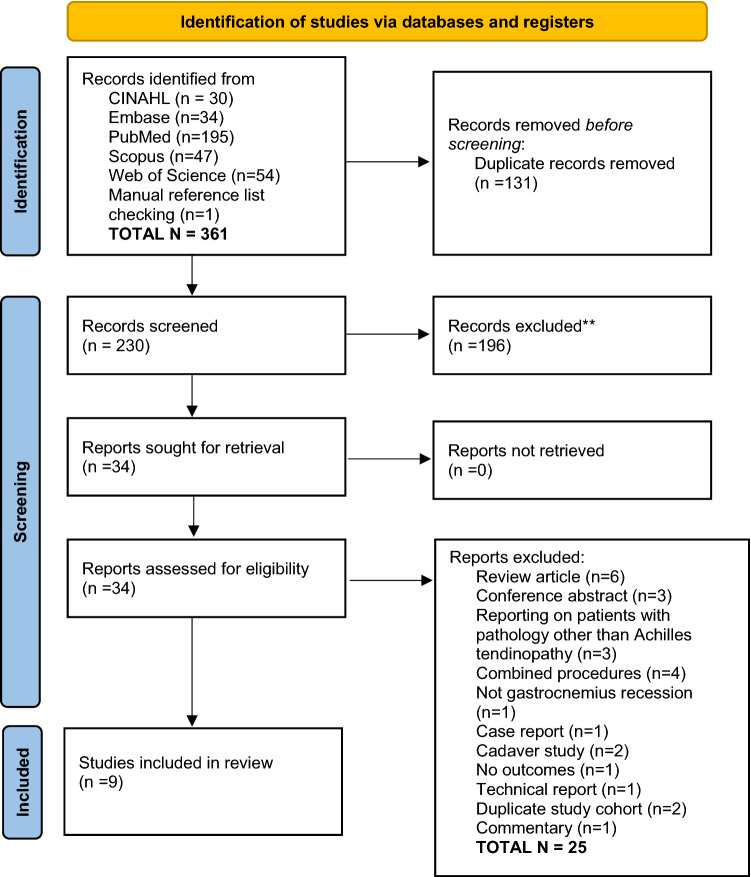
PRISMA flow diagram detailing the number of studies retrieved, removed at each screening stage and included in the final review

The number of studies retrieved during the searching process and removed during the title/abstract and full-text screening stages are displayed in a PRISMA flow diagram (Fig. 1) [39]. Summary study characteristics including number of patients, type of treatment, patient age, patient gender ration and follow-up period are detailed (Table 1). Results are presented according to a qualitative thematic synthesis, focusing on distinct themes identified, such as patient reported outcome measures, complications, ankle range of motion and return to sport/work.

**Table 1:** Table detailing summary study characteristics including study type, number of patients, type of AT, male:female sex ratio, patient age and follow up time

Author	Year	Study type	Number of patients	Type of AT	M:F	Mean age in years (range)	Mean follow up in months (range)	Conservative treatment
DiLiberto [13]	2020	Comparative	8	5 I, 3NI	6:2	51.5 ± 10.2	All at 6 months, 7/8 at 24 months	≥ 6 months
Duthon [14]	2011	Case series	14 (17 legs)	All NI	11:3	42 (20–64)	All at 12 months, 13/14 at 24 months	≥ 1 year *symptoms
Gurdezi [15]	2013	Case series	9	5NI, 4I	4:5	45 (25–63)	30 (20.4–39.6)	6 months- 15 years symptoms*
Kiewiet [20]	2013	Case series	8	All NI	1:7	49.9 ± 11.6	34.6 ± 18.1	≥ 6 months
Maffulli [27]	2015	Case series	18	NR	7:11	46 (34–69)	54 (40–66)	9–28 months symptoms*
Molund [35]	2016	Case series	30 (35 legs)	All NI	9:20	45.5 (14–69)	37.5 (6–93)	≥ 3 months
Nawoczenski [37]	2016	Comparative	14	11 I, 3NI	8:6	52.8 ± 7.9	19.2 (12–28.8)	≥ 6 months
Smith [45]	2018	Case series	25	16 I, 5 NI, 4 both	5:20	53.2 (29–88)	13.1 (3–25)	≥ 3 months
Tallerico [48]	2015	Case series	11	All I	3:8	59 (40–79)	13.8 (12–20)	3–12 months

*These studies describe that all patients failed to respond to previous conservative treatment but did not mention the duration of this treatment and instead report the symptom duration before gastrocnemius recession was performed

*I* insertional Achilles tendinopathy, *NI* non-insertional Achilles tendinopathy, *M:F* Male:female sex ratio, *NR*: Not reported

### Risk of bias and quality of evidence assessment

The risk of bias and quality of the case series and cohort studies included was assessed using the Methodological Index for Non-Randomized Studies (MINORS) criteria [44]. This uses a 12-item checklist, with each item receiving a score of 2 (adequately reported), 1 (inadequately reported) and 0 (not reported). All 12 items are applicable to comparative studies, which can, therefore, receive a maximum score of 24, while non-comparative studies are scored using only the first 8 items of the checklist, with a maximum score of 16 [44].

## Results

A total of 229 articles were retrieved for screening, of which 9 articles meeting all selection criteria were included (Fig. 1). The included articles described a combined total of 145 cases (137 patients) of gastrocnemius recession for Achilles tendinopathy (Table 1). Of the 9 studies, 7 were level IV case series, while two were level III comparative studies, comparing results in patients receiving gastrocnemius recession for AT, with those in healthy controls. The quality of included studies was mixed, with the seven case series studies receiving a median MINORS score of 10/16 (range: 6–12) and the two comparative studies scoring 18/24 and 20/24, respectively (Table 2).

**Table 2:** Table detailing results of the MINORS critical appraisal process

Author	1	2	3	4	5	6	7	8	9	10	11	12	Total
DiLiberto	2	0	2	2	2	2	0	2	2	2	2	2	20/24
Duthon	0	2	2	2	0	2	2	0	NA	NA	NA	NA	10/16
Gurdezi	2	2	2	2	0	2	0	0	NA	NA	NA	NA	10/16
Kiewiet	2	0	2	2	0	2	0	0	NA	NA	NA	NA	8/16
Maffulli	2	0	2	2	2	2	2	0	NA	NA	NA	NA	12/16
Molund	2	0	2	2	0	2	2	0	NA	NA	NA	NA	10/16
Nawoczenski	2	2	2	2	0	2	0	0	2	2	2	2	18/24
Smith	0	2	2	2	0	2	2	0	NA	NA	NA	NA	10/16
Tallerico	0	0	0	2	0	2	2	0	NA	NA	NA	NA	6/16

The numbers in the first row refer to the corresponding item number in the MINORS checklist

*NA* not applicable

All studies reported patients underwent non-operative treatment, such as footwear modification and eccentric stretching exercises, prior to being offered a gastrocnemius recession procedure. A non-operative treatment duration of at least 3 months was described in the six studies reporting this information [13, 20, 35, 37, 45, 48]. Three studies did not report the duration of non-operative treatment, instead reporting a symptom duration of 6 months–15 years (Table 1) [14, 15, 27]. Two studies describe use of an endoscopic technique in 7/11 (63.6%) and 4/35 (12.9%) of cases, respectively, while all other articles utilise an open approach [35, 48] (Table 3). A variety of recession levels are used, with five studies using a distal/Strayer recession procedure, two describing a proximal medial recession and two performing recession at the level of the musculotendinous junction (Table 3).

**Table 3:** Table detailing pre- and post-operative outcome scores for each included study

Author	Procedure details	Mean pre-operative scores	Mean post-operative scores	Significant improvement
Diliberto	Open Strayer Procedure	VAS: 7.1 ± 1.7 FAAM ADL: 68.3 ± 9.6 FAAM sports: 31.9 ± 18.5	VAS: 0.2 ± 0.4 FAAM ADL: 97.4 ± 1.9 FAAM sports: 91.9 ± 7.9	Yes All *P* < 0.01
Duthon	Open release distal to the level of the musculotendinous junction	AOFAS: 71 (67–73) FFI: 39 (25–45) SF-12 physical: 36 (33–34)	AOFAS: 100 (90–100) FFI: 12 (10–19) SF-12 physical: 51 (42–56)	Yes for all AOFAS: *P* < 0.001 FFI: *P* = 0.001 SF-12 physical: *P* = 0.005
Gurdezi*	Open proximal medial release	AOFAS: 69.4 (36–75) VISA-A: 42.4 (12–80) VAS: 8.3 (5–10)	AOFAS: 85.4 (60–100) VISA-A: 84.9 (36–100) VAS: 2.7 (0–9)	Yes *P* < 0.05 for all non-insertional, *P* = 0.05 for all insertional
Kiewiet	Open procedure, distal to the musculotendinous junction	AOFAS: NR FFI: NR SF 36 physical: NR SF-36 mental: NR	AOFAS: 94.4 (57–100) FFI: 7.0 (0–36) SF-36 physical: 81.3 (25–100) SF-36 mental: 68.5 (36–92)	NA
Maffulli	Open release of the medial head, 1.5 cm proximal to musculotendinous junction	VISA-A: 52.3 (no range given)	VISA-A: 75 (51–94) 15/18 (83.3%) rated outcomes as good or excellent according to Boyden classification	Yes *P* < 0.001 for VISA-A
Molund	Open Strayer procedure in 31 legs, endoscopic Strayer in 4 legs	VISA-A: 39.5 (21–51), available in 8 patients only VAS: 7.5 (5–10)	VISA-A: in same 8 patients = 91.9 (68–100), in overall cohort = 91.4 (29–100) VAS: 0.7 (0–7)	Yes *P* < 0.01 for both
Nawoczenki	Open Strayer procedure	VAS: 6.8 ± 1.8 FAAM-ADL: NR FAAM sports: NR	VAS: 1.6 ± 2.3 FAAM-ADL: 90.0 FAAM sports: 70.6	Yes VAS: *P* < 0.05
Smith	Open release at the level of the musculotendinous junction	FFI: 73.5 (32.9–100) VAS:8.9	FFI: 27.4 (0–74) VAS: 1.0	Yes *P* < 0.001 for both
Tallerico	Seven patients endoscopic, four patients open. All at the level of the gastrocnemius aponeurosis	AOFAS: 52.0 ± 4.0 (median)	AOFAS: 94.8 ± 6.3 (median)	Yes *P* < 0.001

*The study of Gurdezi et al. reports outcome scores separately for non-insertional and insertional Achilles tendinopathy. The values presented here are the weighted mean of these two groups. The significance values show are separated according to noninsertional/insertional tendinopathy

### Patient-reported outcome measures

A wide variety of patient-reported outcome measures (PROMs) have been used to assess the effect of gastrocnemius recession for AT, including AOFAS, VISA-A, VAS, Foot function Index (FFI), 36 item short form survey (SF-36) and Foot and ankle ability measure (FAAM) (Table 3). Pre- and post-operative PROMs are detailed (Table 3). All studies, which report both pre- and post-operative PROMs, describe statistically significant (*P* < 0.05) post-operative improvements in at least one of these scores (Table 3). The VISA-A score has a reported minimally clinically important difference score of between 6.5 and 14 points [22, 33]. The three studies reporting VISA-A scores, report mean post-operative improvements of 42.5, 52.4 and 22.7 respectively, all well above the higher end MCID estimate of 14 points [15, 27, 35]. While a wider range of MCID values are suggested for the FAAM activities of daily living (FAAM–ADL) (8–22.7) and FAAM sports (9–32.5) subscales, the one study reporting changes in these scales again reported clinically important improvements of 29.1 (FAAM–ADL) and 60 (FAAM sports) [13, 18, 29, 47]. A similar trend is seen in VAS Pain scores, for which mean post-operative improvements of 5.6, 7.9. 6.9, 5.2 and 6.8 are described (Table 3). Again, this is much higher than the proposed VAS pain MCIDs of 1.4–3. Points [23, 30, 47, 49]. With respect to AOFAS scores, mean post-operative improvements of 42.8, 16 and 29 are described (Table 3). When comparing this to the proposed AOFAS MCIDS of 17–30.2, all but one study failed to reach the higher end MCID estimate [7, 11].

Nawoczenski et al. compared PROMs in patients receiving gastrocnemius recession with healthy controls [37]. Those receiving surgical intervention showed significantly lower mean (*P* < 0.01) FAAM-ADL and FAAM sports score of 90.0 ± 8.4 and 70.6 ± 22.4 compared with 98.3 ± 3.6 and 94.6 ± 10.9 in the control group.

### Range of motion

Two studies compared pre- and post-operative ankle dorsiflexion range of motion [13, 14]. Duthon et al. reported an increase in dorsiflexion from a mean of − 6° pre-operatively to 7° post-operatively [14]. Similar results are seen in the study of Diliberto et al. which describes an increase from 12.2 to 15.9° [13].

### Return to work/sport

Two studies report all patients were able to return to work after operative intervention and a further study reported a return to work rate of 10/11 (90.9%) [14, 27, 48]. Duthon et al. reported that 11/14 (78.6%) of patients were able to return to sport, whilst another study reports that all patients were able to return to the same daily activities as they were able to perform pre-operatively [14]. Gurdezi et al. report that 6/9 (66.6%**)** of patients were able to return to the same or improved level of activity post-operatively [15].

### Factors which may affect treatment outcome

One study performed a subgroup analysis comparing results in those with retrocalcaneal spurs to those without spurs [48]. While both groups showed a significant improvement in AOFAS scores post-operatively, from 51.1 ± 3.9 to 91.9 ± 6.1 (with spurs) and 53.5 ± 4.4 to 100 ± 0.0 (without spurs), the improvement was greater in the patients without spurs [48]. A further study reported that a patient body mass index (BMI) of greater than 30 was associated with decreased post-operative VISA-A scores (*P* = 0.04) [27].

Another potential prognostic factor which authors have investigated is the specific presentation of Achilles tendinopathy. One study found that post-operative improvements in AOFAS, VISA-A and VAS scores reached statistical significance in the noninsertional tendinopathy group, with scores in the insertional group failing to reach the significance threshold (*P* < 0.05) [15]. A similar trend was also described by Smith et al., with greater improvements in VAS and FFI reported in those with noninsertional tendinopathy, compared to insertional tendinopathy [45]. These inter-group differences failed to reach statistical significance.

### Complications

The occurrence of post-operative complications was reported by seven studies describing a total of 123 cases (115 patients) of gastrocnemius recession for Achilles tendinopathy (Table 4) [14, 15, 20, 27, 35, 45, 48]. Sural nerve injury was the most common complication, occurring in 5/123 (4.1%) of patients, whilst the overall complication rate was 10/123 (8.1%).

**Table 4:** Table describing the incidence of complications reported across all included studies

Author	Number of feet	Wound healing	Nervous system	Other	Total
Tallerico	11	–	2 sural nerve paraesthesia	1 recurrence of insertional heel pain and equinus deformity	3/11 (27.3%)
Kiewet	8	–	–	–	0/8 (0%)
Gurdezi	9	1 hypertrophic scar	–	1 DVT	2/9 (22.2%)
Smith	25	–	2 sural neuritis	1 distal Achilles rupture 4 months post–operatively (unknown if related to operation)	3/25 (12%)
Molund	35	1 wound infection	1 sural nerve injury	–	2/35 (5.7%)
Maffulli	18	–	–	–	0/18 (0%)
Duthon	17	–	–	–	0/17 (0%)
Total	123	2/123 (1.6%)	5/123 (4.1%)	3/123 (2.4%)	10/123 (8.1%)

*DVT* deep vein thrombosis

## Discussion

This study aimed to systematically review and summarise the outcomes of gastrocnemius recession for Achilles tendinopathy. A total of nine studies meeting all selection criteria were included, with the majority of studies being level IV case series and only two level III comparative studies included.

### Patient reported outcome measures

The only PROM which has been specifically validated for Achilles tendinopathy is VISA-A, which does show post-operative improvements much greater than the range of proposed MCID scores [42]. Furthermore, both the FAAM and FFI scales, for which general validation has been demonstrated in foot and ankle disorders, and the VAS pain score, which is validated in the measurement of acute and chronic pain, showed post-operative improvement consistently greater than the MCID [4, 5, 29]. These results suggest that gastrocnemius recession is a beneficial treatment option in patients with Achilles tendinopathy. On the other hand, of the three studies reporting change in AOFAS score, two reported statistically significant, yet, sub-MCID improvements (Table 3). However, it is important to interpret these scores with caution. The AOFAS now recommends against use of this score due to factors such as a small number of response intervals leading to limited precision and a skewed distribution [2, 16, 40].

These promising results must be interpreted in light of the risk of bias of included studies. While all studies use an appropriate follow-up period, likely to allow the assessment of all relevant benefits and harms, only 5/9 studies reported a loss to follow up of less than 5% (Table 2). The remaining four studies report loss to follow up rates of 12.5–33.3%, introducing a significant degree of transfer bias. Furthermore, a number of studies do not specify inclusion of consecutive patients, which may introduce selection bias (Table 2). Assessment bias must also be considered as all but two studies failed to describe unbiased or blinded assessment of study outcomes. Together, these biases are likely to inflate the apparent treatment benefit described. However, given our results show improvements consistently greater than MCID across many PROMs, it is likely that even considering these biases, and there is a clinically important treatment effect.

With the exception of Maffulli et al. and Kiewet et al. all studies only included patients with gastrocnemius contracture [20, 27]. Therefore, while the evidence suggests gastrocnemius recession is effective in patients with AT and associated gastrocnemius contracture, it is not clear what proportion of patients with AT suffer from gastrocnemius contracture and whether recession provides any benefit in patients without contracture. Until this becomes clearer, the current evidence supports gastrocnemius contracture only in patients with an associated gastrocnemius contracture. Assessment of contracture should therefore be performed to aid clinical decision making in all patients with AT for whom surgical intervention is being considered.

### Factors which may affect treatment outcome

This review identifies some potential factors associated with a poorer outcome, such as the presence of a heel spur, BMI greater than 30 and insertional Achilles tendinopathy [27, 45, 48]. However, this evidence is derived from single studies with relatively small cohorts and a significant degree of bias. Further research into identifying patient related factors that may affect treatment outcomes is required before clinicians can use these to stratify patients for the purposes of clinical decision making. This will also aid in the refinement of selection criteria for gastrocnemius recession and the selection of the most appropriate surgical intervention.

### Complications

A previous systematic review investigating a variety of surgical interventions for AT reported overall complication rates of 5.3% (endoscopic surgical techniques) and 10.1% (open surgical techniques). This is similar to the complication rate of 10/123 (8.1%) reported in this review [25]. Many of the complications described following gastrocnemius recession are common peri-operative complications such as wound infection, hypertrophic scaring or DVT, which may occur following any surgical procedure [12]. One specific concern regarding gastrocnemius recession, particularly in distal recession techniques, is the incidence of sural nerve injury [6, 32, 36]. However, our review shows that while sural nerve injury was the most common complication, the overall incidence remained relatively low at 5/123 (4.1%) cases. Three of these cases occurred following use of an endoscopic recession technique. Although the incidence of sural nerve injury is low, clinicians should take extreme care to identify and protect this nerve during the procedure.

## Conclusions

This systematic review demonstrates that gastrocnemius recession is a good treatment option in patients with Achilles tendinopathy who suffer from gastrocnemius contracture and who have failed to respond to non-operative management. Postoperative changes in PROMs such as VISA-A, VAS pain, FFI and FAAM are consistently above the MCID., Complications are reported in approximately 8% of patients, with most of these being minor complications which may be seen following any surgical intervention.

## Data Availability

Available on request.

## Code availability

N/A.
